# Mucus, Microbiomes and Pulmonary Disease

**DOI:** 10.3390/biomedicines9060675

**Published:** 2021-06-13

**Authors:** Oliver W. Meldrum, Sanjay H. Chotirmall

**Affiliations:** Lee Kong Chian School of Medicine, Nanyang Technological University, Singapore 308232, Singapore; oliver.meldrum@ntu.edu.sg

**Keywords:** mucus, mucin, microbiome, pulmonary disease

## Abstract

The respiratory tract harbors a stable and diverse microbial population within an extracellular mucus layer. Mucus provides a formidable defense against infection and maintaining healthy mucus is essential to normal pulmonary physiology, promoting immune tolerance and facilitating a healthy, commensal lung microbiome that can be altered in association with chronic respiratory disease. How one maintains a specialized (healthy) microbiome that resists significant fluctuation remains unknown, although smoking, diet, antimicrobial therapy, and infection have all been observed to influence microbial lung homeostasis. In this review, we outline the specific role of polymerizing mucin, a key functional component of the mucus layer that changes during pulmonary disease. We discuss strategies by which mucin feed and spatial orientation directly influence microbial behavior and highlight how a compromised mucus layer gives rise to inflammation and microbial dysbiosis. This emerging field of respiratory research provides fresh opportunities to examine mucus, and its function as predictors of infection risk or disease progression and severity across a range of chronic pulmonary disease states and consider new perspectives in the development of mucolytic treatments.

## 1. Introduction

Pneumonia remains one of the leading causes of morbidity and mortality globally, with nearly 2.3 million deaths reported in 2016. It disproportionately affects the elderly, individuals with comorbidities and children under 5 years of age [1,2,3]. Pneumonia, irrespective of underlying aetiology, associates with significant airway inflammation including mucus accumulation within the lung’s airspace. Understanding microbial aetiology in respiratory infections such as pneumonia is evolving with new, rapid molecular next-generation sequencing approaches highlighting unculturable pathogens and co-infection at higher frequencies [4]. While any individual may develop respiratory infection and/or pneumonia, risks are higher in those with chronic airways disease such as asthma [5], cystic fibrosis (CF) [6], non-CF bronchiectasis [7], and chronic obstructive pulmonary disease (COPD) [8,9], whose disease course is punctuated by episodes of recurrent infection and exacerbation which, in turn, increases mortality risk.

As a warm, moist environment, the airway is colonized by distinct microbial populations even in the stable and healthy state with varied composition from upper to lower airways [10]. Considered one of the key lung defenses against infection, mucus acts as a physical barrier by coating the respiratory tract (bar the terminal bronchioles and alveoli) to entrap microbes and facilitate their extrusion through mucociliary clearance and the cough reflex [11]. While critically important, this is a limited conceptualization of the greater role that mucus plays in protecting the lung; for instance, it fails to explain how mucus accommodates the stable coexistence of a diverse resident lung microbiome [12]. Healthy lungs contain a highly diverse inter-kingdom community of bacteria, viruses, and fungi including *Prevotella*, *Veillonella*, *Streptococcus*, *Haemophilus*, *Neisseria*, and *Corynebacteria* [13,14,15]. A healthy lung mycobiome is described and the role of microbial interactions within the lung in disease pathogenesis and progression is emerging [16,17]. How this relates to resident mucus and its properties remains to be elucidated. Changes in the physicochemical properties of mucus during active infection and/or exacerbation, driven largely by inflammation, further impair microbial clearance while enhancing the growth of particular resident microbes that may develop into pathobionts [18]. The airway microbial community, particularly in the setting of chronic airways disease, changes over time, demonstrating a reduced diversity typically associated with inflammation [19], antimicrobial use [20], and worsening lung function [21]. Important and additional insight may be gained through studying the mucus–microbiome relationship: it may offer improved non-invasive approaches to patient risk stratification or provide targeted opportunities for treatment independent of antimicrobial therapy [22]. Here, we review and discuss the properties, function, and clinical translational relevance of pulmonary mucus and assess it as a potential treatment target in pneumonia and chronic pulmonary disease.

## 2. The Structure and Function of Pulmonary Mucus

The surface of intrapulmonary airways is dominated by ciliated and secretory cell types [23]. The latter produce mucus of differing composition and are subtyped based on microscopic appearance including serous, neuroendocrine, mucin-[goblet], and club cell secretory protein (CCSP)-expressing secretory [club] cells [24]. Airway mucus represents a multi-component secretion best described as a biological hydrogel composed of water, polymerizing mucin glycoproteins (MUC5B and MUC5AC), a range of antimicrobial molecules (defensins, lysozyme, etc.), cellular components (cellular debris including DNA and keratin), and protective factors (trefoil factors) [25]. All such components are perpetually synthesized, secreted, and integrated into mucus before appropriate degradation and clearance. MUC5B and MUC5AC, the major polymerizing mucin glycoproteins, are essential to normal mucus clearance from the human airway. In healthy airways, MUC5B remains the dominant secretory mucin in submucosal glands and superficial airway epithelia, while MUC5AC is predominantly produced in superficial epithelia lining the proximal (cartilaginous) airways [26]. Importantly, neither is expressed in the terminal bronchioles, suggesting that gas exchange requires a surfactant-rich, mucin-free zone to protect adjacent alveoli.

### 2.1. Polymerizing Mucins

**Figure 1:**
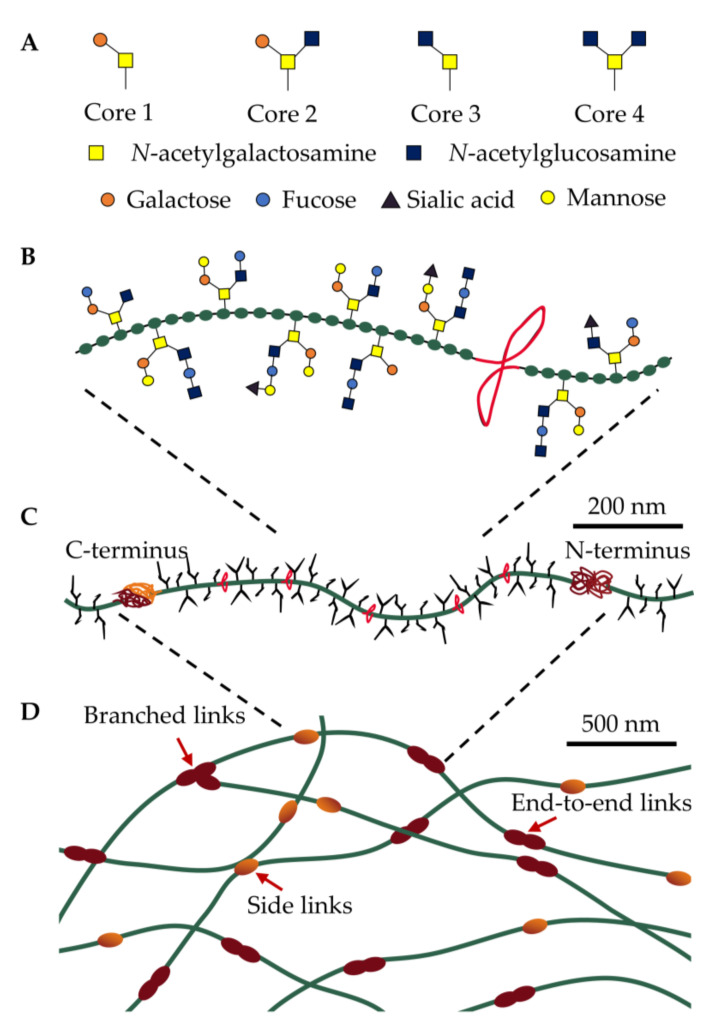
The polymeric and macromolecular structure of polymerizing airway mucins. (**A**) The mucin backbone is the primary site of O-linked glycosylation (Core 1–4) that attach to (**B**) serine-, threonine-, and proline-rich amino acids (green). These regions are interspersed with cysteine-rich regions (CysD domains, red) containing hydrophobic amino acids and intramolecular disulfide bonds. (**C**) The mucin monomer contains C- and N-termini as sites of intermolecular disulfide bonds that (**D**) assemble into multimer states that exist as linear, branched, or side-linked structures. The mucus gel is a dynamic network, subject to removal and reformation of chemical and physical interactions via disulfide bonds and entanglement from low-energy electrostatic and hydrophobic interactions, respectively.

Mucins are a family of molecules composed of polymerizing and non-polymerizing forms of secreted and cell-tethered mucins. The focus of this review is airway-secreted mucin, in particular its polymerizing forms. The secreted, polymeric mucins in the airways are characterized by a protein core organized into two regions, a central linear region dominated by proline-, serine-, and threonine-rich amino acids and a second amino- and carboxyl-terminal globular region (Figure 1C). The central region undergoes O-linked glycosylation (hereafter referred to as glycans) and accounts for up to 80% of mucin’s molecular weight forming negatively charged hydrophilic zones that protect the protein core from protease degradation through a linear, “bottlebrush”-like structure [25]. Each glycan respectively contains 2–20 neutral and/or negatively charged sugars that are initiated by the addition of *N*-acetylgalactosamine (GalNAc) to the hydroxyl group of serine- or threonine-resides. These *O*-glycan structures present in mucin are diverse and complex, consisting predominantly of a core 1-4 mucin-type consisting of GalNAc, galactose and *N*-acetylglucosamine (Figure 1A). These core structures are further elongated and modified by the addition of fucose and sialic acid, or small amounts of mannose and sulfate (Figure 1B) [27]. The complexity and diversity of mucin glycans, therefore, offers a high degree of resistance to microbial protease, glycosidase, and sialidase activity, due to the range of specific enzymes needed to degrade the plethora of glycan linkages.

Polymerizing mucins possess a unique form of hierarchical supramolecular assembly, where monomers with molecular masses of up to 2.5 mDa assemble into a matrix interconnected by chemical cross-linkages and physical interactions (Figure 1D) [28]. Non-mucin molecules including proline-rich proteins, trefoil factor-1, and keratins likely further contribute to mucin assembly, although their roles have not been fully expounded [29,30]. These multiple levels of assembly at different length scales result in a unique set of physicochemical and mechanical properties, where fluid-like (viscous) and solid-like (elastic) behavior are required to be in balance to ensure normal physiological function. At the macroscale, mucus behaves as a viscoelastic gel (G′ > G″), while at the microscale, mucus is composed of viscoelastic micro-domains that assemble into a yield stress fluid [29]. Our ability to adequately describe the complex rheological (flow) properties of mucus is limited when using simplified terms such as viscosity or viscoelasticity, which omits other aspects of its complex supramolecular dynamics including time-dependent flow (e.g., creep), hysteresis behavior, spatial and compositional heterogeneity. There is an increasing interest in understanding the relationship between mucus composition, viscoelastic properties, and transport within airways [31,32]. As rheological properties of mucus gels largely depend on the degree of covalent cross-links among mucins [29], mucus pathology is likely a result of defective swelling, where small changes in swelling equilibrium result in significant consequences for mucus rheology. Consequently, the pathophysiology of defective mucus remains largely phenomenological and an understanding of mechanisms that allow the transport of airway mucus remain limited.

**Figure 2:**
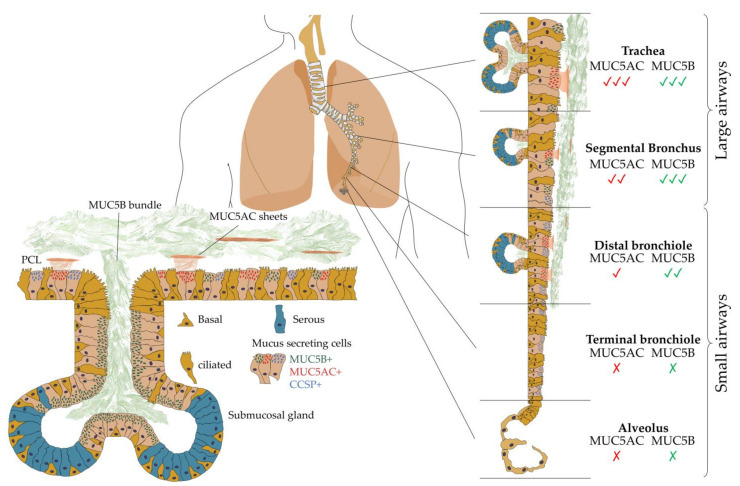
Airway localization of polymerizing mucins. Regional distribution of MUC5AC (red) and MUC5B (green) mucin glycoproteins along the proximal–distal axis of normal airway epithelia with neither MUC5AC nor MUC5B expressed in the terminal bronchioles or alveoli. MUC5B (MUC5B+) is the dominant secretory mucin in submucosal glands and superficial airway epithelia, while MUC5AC (MUC5AC+) is secreted into the superficial epithelial lining in the proximal airways and locates alongside non-polymerizing mucin club cell secretory protein (CCSP+)-expressing cells. These polymerizing mucins overlay the periciliary layer (PCL) and are propagated by mucociliary action out of the lungs. Adapted from data presented in [26].

Mucus is separated from the airway’s epithelial surface by a periciliary layer (PCL), an approximately 200 nm inter-ciliary space densely occupied by membrane-spanning mucins and acidic mucopolysaccharides tethered to airway cilia, microvilli, and the epithelial surface. MUC5B mucin, produced by submucosal glands, form linear extended bundles with a diameter of 20–30 µm, which transiently couple to the more superficial airway epithelia-derived MUC5AC [33] (Figure 2). A ‘Gel-on-Brush’ model has been proposed to illustrate how membrane-spanning mucins and mucopolysaccharides act as a surface lubricant, preventing the adhesion of polymerizing mucins whilst allowing the formation of a distinct mucus layer over the airway surface epithelium [34]. Cilia-driven mucus flow may therefore facilitate “mucus swirls” which contain localized regions of varying mucin concentration, allowing smaller molecules to rapidly diffuse through while larger particles and infectious agents such as bacteria may become entrapped [29,35,36].

The mucosal response to bacterial incursion remains dominated by goblet-cell-driven microbial sensors that initiate the secretion of polymerizing mucins, entrap invading microbes, and release a dilute mucus bolus in attempts to “flush” bacteria away through mucociliary clearance [37]. MUC5B represents the dominant mucin in healthy mucus, emerging from submucosal glands and containing bundle structures composed of multiple filaments necessary to sustain mucociliary clearance [38,39]. In contrast, MUC5AC (and some MUC5B) are secreted from superficial goblet cells, forming thin threads that coalesce into sheets. Such responsive action of MUC5AC has gained its reputation as a “response mucin” owing to its ability to provide a critical innate immune function during times of airway stress [40,41,42]. Taken together, both MUC5B and MUC5AC contribute, albeit differently, to mucociliary clearance in the healthy lung, where the latter further contributes to an inappropriately hyperconcentrated mucus that may obstruct the airway [34,37]. Paradoxically, while a deficient mucus layer may leave the lungs vulnerable to injury, hyperconcentrated mucus or an impaired mucus clearance may also contribute to disease pathogenesis and progression across a range of pulmonary diseases (Table 1). Mucus percent solids (complex mixture of biomolecules and inorganic salts) [43], extracellular DNA [44], and an overproduction of respiratory mucins, especially MUC5AC, confers mucus stasis and a positive feedback cycle of inflammation, further mucus accumulation, and lung damage. MUC5AC and MUC5B quantification are also highly variable between studies, due to the differing biochemical assays employed, and the rapid and highly variable degradation that occurs in the proximal airway before sputum expectoration further complicates such measurements [45].

### 2.2. Translating Mucin Properties into Understanding Pulmonary Disease

Airway mucin hyperconcentration is related to respiratory symptoms across a range of pulmonary disease states and represents a key target for the development of new therapeutics. A clear opportunity to use airway mucins as a potential predictor of individuals at increased risk of more symptomatic and/or exacerbating disease exists. Importantly, before we can realize such potential, there remain fundamental gaps in our knowledge and understanding of airway mucin structure, including its function in both the healthy and diseased airway. Further studies to better understand mechanisms through which mucins directly contribute to the development of mucus abnormalities, airway inflammation, infection, and the progression of airflow obstruction are required: for example, how different mucins and their glycan side chains control gel-formation and whether these are linked to respiratory disease pathology. It remains important to appreciate the role that mucus has in the host–commensal and host–pathogen microbial relationship. In particular, how the host maintains such a complex microbial community in the lung and the specific changes that occur during pathogenic infection including the role, function, and compositional change in mucus. Improved knowledge of the basic aspects of mucin biology and their alteration in response to infection will better inform prospective strategies to alleviate mucin hyperconcentration and obstruction in acute and chronic pulmonary diseases (Table 2). Developing this knowledge may reveal novel aspects of mucus that can be employed to improve diagnostics and/or risk stratification in respiratory disease states that suffer from significant patient heterogeneity.

## 3. Going Beyond Mucus as a Barrier: The Role of Mucin in Bacterial Pathogenesis and Chronic Respiratory Disease

The gut microbiome including its role in regulating host metabolism is well studied; however, gaps exist in understanding the identity, abundance, and functionality of the lung microbiome in the setting of chronic respiratory disease [12,59]. Mucus-dwelling microbes form metabolic niches through resilient associations within a complex community that derives nutrition from host mucin glycoproteins in the lung [60]. Only a few bacterial species contain a sufficient repertoire of genomic-encoded catabolic glycosidic enzymes to disassemble complex mucin glycans as a carbon source, highlighting the existence of a cross-feeding cabal, akin to the role of microbiota in the gastrointestinal tract [61,62]. The lung microbiota has now been shown to have important roles in lung development [63] and maintaining homeostasis [64,65]. For instance, bacterial metabolites including glycolic acid, indol-3-acetate, and linoleic acid blunt the alveolar macrophage response to endotoxin challenges in COPD patients with emphysema [66]. This raises a key question of whether microbes within mucus adopt specialized metabolic, spatial, and proliferation adaptation strategies to persist, and if so, what dysfunction occurs in the setting of pulmonary disease and how this contributes to pathogenesis and progression?

### 3.1. Mucin as a Nutritional Source

In addition to their protein backbone, mucins contain hundreds of unique glycan structures that require the action of specific glycosidases to be broken down and utilized as energy sources [67,68]. For such breakdown, complex microbial communities benefit from interspecies interaction, including metabolic partitioning that requires cooperation between individual bacteria to promote community stability. A complex set of mucin glycans offers a diverse nutrient reservoir, enabling the host to support even the most complex of microbial communities [69]. In contrast, limited glycan diversity may result in inefficient energy harvest that leads to an outgrowth of a small (pathogenic) microbial population.

One of the key causes of pneumonia, *Streptococcus pneumoniae*, possesses key virulence mechanisms to degrade, transport, and metabolize a range of complex host glycans [70,71,72], therefore disrupting healthy resident microbial communities [73]. In contrast, most other respiratory pathogens have relatively restricted glycan utilization capabilities, which require a polymicrobial community of cross-feeding microbes to facilitate their growth. For instance, *Pseudomonas aeruginosa* is unable to utilize mucin glycans in isolation, requiring secondary metabolites generated by human-host commensals such as short-chain fatty acids and amino acids to sustain its survival and promote growth [60]. Co-cultivation of two important respiratory pathogens causing pneumonia, *Staphylococcus aureus* and *P. aeruginosa*, is shown to enhance *P. aeruginosa* persistence even in the presence of gentamicin and tetracycline antibiotics [74,75]. Individual mucin-derived monosaccharides enhance *P. aeruginosa* virulence in *C. elegans* and human cell lines [76], suggestive that such increased pathogenicity may at least in part be attributed to their ability to metabolize glycans and share the derivatives with other co-existing species.

### 3.2. Mucin Permits the Spatial Organization of Lung Microbial Communities

While the primary function of polymerizing mucin lies in its ability to transport microbes away from the epithelium, mucin “networks” can be considered alternate extracellular matrices, likened to bacterial biofilms, that provide a three-dimensional scaffold mediating the spatial organization of beneficial microbial communities in the lung. Such a structure contributes to the localized selection and maintenance of healthy commensal organisms while controlling the outgrowth of harmful pathogens [77]. During pulmonary infection, microbial dysbiosis leads to opportunistic pathogens-forming biofilms, where cells aggregate within an extracellular matrix protected from competition [78]. These communities disrupt particular tissue compartments including mucus within the airway lumen causing progressive, localized, and chronic infection, particularly in pulmonary diseases. During a period of stability, patients with CF demonstrate a polymicrobial lung milieu, where *P. aeruginosa* produces antimicrobial products in an attempt to exclude *Burkholderia cepacia* (*Bcc.*) from forming biofilm-like structures [79]. During an exacerbation event, however, anaerobic conditions permit *Bcc.* to invade *P. aeruginosa*-infected CF lungs, outcompete other microbes through anaerobic fermentation and the metabolism of mucin [80]. This results in *Bcc*.-infected CF lungs, which, in turn, have clinical consequences including diminished long-term survival [81]. Such phenomena indicate that it is not a biofilm-phenotype per se that drives lung disease in the setting of CF and other chronic pulmonary disease states, but the metabolic and functional disruption of pulmonary mucin that significantly contributes to disease progression.

### 3.3. Mucin Signaling and Microbial Behavior in the Lungs

While mucins serve as a nutritional source and support microbial communities within its structure, polymerizing mucin (i.e., MUC2, MUC5AC, MUC5B, MUC6, and MUC19) across different body sites illustrate a remarkably broad ability to attenuate microbial virulence in evolutionarily distinct pathogens [82,83,84,85]. Evidence that mucin contributes to microbial coexistence exists, predominantly through providing a trigger for the downregulation of virulence genes involved in quorum sensing, toxin secretion, and the regulation of biofilm formation: all hallmarks of mucosal infection [77,83]. Several studies propose that mucin directly pacifies harmful pathogens into host-compatible commensals by suppressing their virulence, thereby contributing to the coexistence of opportunistic pathogens within the mucus environment [86,87]. Mucin glycans are potent host-derived manipulators of the bacterial phenotype by downregulating virulence pathways, for instance, in *P. aeruginosa*, [83] where they promote bacterial disassembly from the outer layers of pre-formed biofilms [88]. This phenotypic switch is triggered by the O-linked mucin glycans to reduce cytotoxicity to human epithelia in vitro and attenuates infection using a porcine burn model. Mucins are also shown to suppress *Candida albicans* virulence, a prevalent fungal commensal which can become pathogenic in appropriate settings, by downregulating a range of genes related to adhesion, penetration, hyphal, and biofilm formation [82].

These findings have broad implications for improving our understanding of how the host delicately balances the control of infection with the maintenance of a diverse, beneficial commensal microbiota. Mucin-associated glycans interact with specific microbial surface adhesins by competing directly against native pathogen polysaccharides, preventing their aggregation and biofilm formation. Currently, >260 glycan structures have been identified, and which of these serve as potential virulence-attenuating signaling molecules remains unknown [89]. It is conceivable that specific glycan structures impact specific virulence pathways in distinct pathogens [89]. While commensal microbes are generally thought to be “host-compatible”, a clearer picture is emerging of the specific role mucin and its associated glycans play in attenuating opportunistic pathogens. However, the actual processes by which commensals populate mucus remains poorly understood. Of particular interest is why diseased mucin no longer retains the ability to attenuate microbial virulence and pacify opportunistic pathogens, particularly during pulmonary infections such as pneumonia. While this may be potentially explained by a reduced O-linked glycan size and change in sulfation and sialylation compared to healthy mucus [90], the loss of ability to attenuate diverse microbial communities in the context of chronic respiratory disease states represents a key challenge to managing and treating pulmonary disease [91].

### 3.4. Mucin in Chronic Pulmonary Disease

Central to the pathophysiology in several pulmonary diseases remain airway inflammation, mucin hyperconcentration, and airway obstruction. Excess mucin associates with several pathological features of asthma, CF, non-CF bronchiectasis, and COPD, resulting in an increased frequency and duration of exacerbations, reduced lung function, and increased morbidity and mortality [92,93]. A COPD patient with chronic bronchitis demonstrates lower bacterial diversity, higher risks of pneumonia, and steeper declines in lung function compared to individuals who produce less phlegm at baseline [92,94,95]. The switch from “healthy” to “diseased” mucus occurs through multiple and concurrent mechanisms that alter its hydration, chemical constituents, and physical properties, including the abnormal secretions of electrolytes and water, mucin production including composition, neutrophilic inflammation, and bronchovascular permeability [30]. An overproduction and impaired clearance can further lead to thick and viscous mucus in the airways, which associates with higher infection rates [53]. Physical signs of impaired mucus clearance can include cough, bronchial breath sounds, rhonchi, and wheeze, which may appear in chest radiographs and on computerized tomography (CT) examination as a septal thickening, nodules, or areas of ground-glass opacification [96,97].

In CF, genetic mutations in the CF transmembrane conductance regulator (CFTR) protein results in reduced chloride secretion and sodium hyperabsorption [98]. Together, this leads to dehydration and an abnormally thick mucus that is difficult to clear, creating a cycle of inflammation, infection, and injury [99,100,101]. Elevated mucus solid concentration and higher partial osmotic pressure leads to an osmotic compression of the PCL, contributing to mucociliary stasis [102]. In the lungs of infants with CF, mucus plaque formation (composed predominantly of MUC5AC and MUC5B) can be seen in greater numbers compared with non-CF pulmonary infection and closely correlates with neutrophilic inflammation and associated products of necrosis such as neutrophil extracellular traps (NETs) [53]. This suggests that bacterial infection may not be primarily responsible for the earliest stages of CF-associated lung disease, highlighting how alterations in mucus composition and physical properties, independent of infection, can initiate lung damage. Mucus plaques and stasis blanketing airway surfaces further increase the susceptibility of the lung to microbial colonization and infection, which result in dense bacterial aggregations at the epithelial surface that are resistant to immune clearance and antibiotic treatment [103,104]. These concepts are reinforced by Hoegger et al. [105], who illustrated that the abnormal tethering of mucin to submucosal glands blocks mucus clearance and Cl^−^ and HCO^3−^ ion secretion in a piglet CF lung model [106]. A single-nucleotide polymorphism in the *MUC5B* promoter (rs35705950) is shown to reduce multiple measures of acute respiratory events, a major driver of COPD mortality in the COPDGene cohort of smokers [107]. The key findings include an almost two-thirds lower risk of prospectively reporting an exacerbation event, 40% fewer acute respiratory events, and a longer timeframe to first event.

Asthma and COPD both associate with significant airway inflammation of the mucosa; however each has distinct clinical entities, inflammatory markers, mediators, and response to therapy [108]. IL-13 is demonstrated to induce alteration in mucin composition and organization [109,110], resulting in increased adherence and impaired mucociliary clearance despite the presence of intact ciliary beating and a PCL [111]. Such impaired clearance is, at least, in part promoted by neutrophilic oxidative stress, leading to increased mucin polymerization and sputum elasticity [112]. During an exacerbation event, mucin overproduction and its associated inflammation leads to airway obstruction, contributing to poorer lung function. Detecting and quantifying mucus plugs using multidetector CT in asthma and COPD is linked to airflow obstruction and poorer lung function outcomes [113,114]. Importantly, the prevalence of mucus hypersecretion is similar in patients with high and low mucus plug scores, highlighting the difficulties in using symptoms as a main surrogate for disease classification. Further studies are therefore warranted to better elucidate changes that occur to mucus before, during, and after acute exacerbation events.

While a potential role for mucin glycan metabolism may exist in the pathogenesis and progression in several pulmonary diseases, several questions regarding nutrient acquisition during late-stage chronic pulmonary disease need exploring. In chronic airway disease, a reduced microbial diversity is observed during disease progression, with Proteobacteria-dominant microbiomes associated with reduced lung function, more frequent exacerbations, and increased mortality [115,116,117]. During inflammation, mucin glycans exhibit a reduced chain length, sulfation, and fucosylation, and increased sialylation [90]. This is observed through *P. aeruginosa* and *H. influenzae* virulence, which induces MUC5AC expression while impeding mucin glycosylation and sulfation [118,119]. The sialylation of airway mucin enhances *P. aeruginosa* adhesion to the airway, facilitating increased colonization and the development of lung disease [91,120]. Dysregulation of mucin-derived glycans also benefits pathogens during infection episodes with some glycans enhancing the binding and internalization of *P. aeruginosa* to human lung epithelia [76]. Such changes, through less diverse glycan sources as nutrients, can lead to the eradication of host commensals, decreasing microbial diversity and its spatial organization.

## 4. Targeting Mucin in Pulmonary Infection and Disease

The increasing burden of pulmonary infection and disease globally necessitates novel and more targeted approaches to treatment to improve patient outcomes. In line with this, a promising and emerging area is the study of mucin. Utilizing mucin status for instance to predict the risk of disease severity, progression and prognosis would be a valuable tool and the mucus complex further affords potential novel opportunities in the treatment of asthma, CF, non-CF bronchiectasis, and COPD. Direct clinical application will require detailed, controlled, and longitudinal studies across a range of disease endophenotypes to better understand if quantifying chemical and physical properties of airway mucins can suitably predict those at greatest risk of disease, symptoms, and progression. For instance, airway mucin concentration is a key component in the pathophysiology of the chronic bronchitis cascade that results in excess sputum production and disease severity [56]. Greater knowledge of mucus composition and relevance to clinical outcomes may be leveraged to better identify patients with higher risks and poorer prognosis allowing for more targeted intervention.

In pulmonary medicine, a long-standing need exists to treat excess mucus accumulation in the airways, a key pathologic feature in a variety of disease states. The overarching goal of inhaled therapies to address this is to clear hyperconcentrated, adherent mucus that causes airflow obstruction, inflammation, and subsequent infection. However, our ability to treat pathologic mucus continues to be hindered by a lack of understanding the mechanisms involved in mucus production and function within infected and/or diseased airways. Additional intermolecular disulfide bonding occurs under inflammation and oxidative stress [112,121], resulting in limited mucus clearance, and a motivation for mucin-targeted mucolytic agents. Reducing agents including Mucomyst (*N*-acetylcysteine [NAC]), a thiol-based drug used across several obstructive pulmonary states, disrupts intermolecular mucin covalent disulfide bonding, which, in turn, reduces the molecular mass of its polymerizing aggregates, enabling easier clearance [122]. Importantly, current clinical data in COPD does not support the efficacy of inhaled NAC in terms of improving lung function and/or reducing COPD exacerbations [123,124]. In contrast, recombinant human DNase (rhDNase), an enzyme facilitating the breakdown of extracellular DNA has shown substantial clinical benefits in adults with CF, including improvements in lung function [125,126,127,128]. Critically, while benefits in CF were clear, the same agents have shown mixed or even harmful effects in other obstructive lung diseases including COPD and non-CF bronchiectasis. In the latter, an increased exacerbation frequency is observed with therapy outlining the importance of understanding the differing pathophysiology of each disease [129]. Recently, Erdosteine, another thiol-based drug containing two blocked sulfhydryl groups that are released following first-pass metabolism in the liver, has been shown to significantly reduce the risk of acute COPD exacerbations, shorten their duration, and decrease hospitalization risks [130]. Newer targets in this space should focus on enabling mucus clearance, targeting the submucosal glands, anion secretion, and mucus maturation. Each respective respiratory disease state likely warrants its own unique “mucus” development pipeline to account for the intricate differences in pathophysiology and disease course.

Due to the inherent interdependency between host, mucus, and resident lung microbes, it is unsurprising that pulmonary disease can be modulated, particularly early in life, by microbial infection and therapy [131,132]. However, different chronic pulmonary disease states demonstrate important differences in “pathological” mucus and treatment; therefore, they may need to be tailored accordingly. While conceptually plausible, simply reducing mucus secretion alone appears relevant, but may not be wholly effective due to the adverse consequences of inadvertently eliminating MUC5B, which in mouse models has shown to promote mucus accumulation and chronic infection [38]. Understanding how particular species- and/or strain-level functional differences in microbial interaction with host immunity may better explain disease status and reveal appropriate individually tailored targets. Dietary probiotics reduce the incidence of ventilator-associated pneumonia, without impacting the duration of mechanical ventilation, length of hospitalization, or mortality [133]. This raises important questions of a potential to partly restore “diseased” respiratory microbiomes using microbiota-directed or host-targeted therapies such as probiotics and/or metabolite supplementation. To date, several studies have attempted to investigate the efficacy of probiotics in asthma [134,135,136] and multi-drug resistant lung infections [137], and while these have produced contradictory results, the increasing awareness of host-associated factors that determine how an individual responds to probiotic treatment has been gained [138].

Despite our current understanding, we still have a very limited appreciation of features specifically relevant to the supramolecular assembly of mucins and their role in mucus dysfunction. The formation of mucin sheets and bundles is observed, but how this unique polymer assembly confers its functional role is incompletely understood. Further, we know little regarding the airway supramolecular assembly process including the programmed unfolding of large mucin chains to allow transition from its condensed structure within a goblet cell to the polymerizing insoluble mucin plaques observed in airway diseases such as CF. While an increased prevalence of opportunistic pathogens is associated with poorer lung function and reduced microbial diversity, we have yet to fully understand how a diseased lung allows certain pathogens to dominate: is it a malfunction in nutritional source for microbes, or perhaps a change in spatial mucus organization or loss of virulence-attenuating signals, or a combination of such factors? Developing the ability to control access to mucin glycans and their derivatives may provide a fresh approach in preventing, controlling, or even disrupting lung infection. In particular disease states, pathogens can establish an infection that results in enhanced binding to the epithelial surface because of a change to mucin glycan composition. Therapeutics controlling mucin expression or glycosylation to diminish pathogen binding or alter its virulence may have value in addressing complex polymicrobial lung infections. Further research is needed to better characterize differences between healthy and diseased mucus including how the surrounding environment influences its properties and function. An understanding of how mucin status is altered before and during exacerbation events will be key and should be pursued.

## 5. Conclusions

While progress has been made, much remains to be explored to fully understand and predict the complex dynamics of mucus in the context of respiratory infection and chronic respiratory disease. Increased mucin concentration coupled to pathogen outgrowth and neutrophil infiltration, creating a unique extracellular environment in pulmonary disease. In this review, we highlight the intricate role mucins play in the host response to pathogens by providing a nutritional source as well as a structural surface for the resident microbial community. Mucus directly influences microbial behavior, although much work remains to elucidate specific mechanisms. Such properties select for and accommodate a diverse yet specialized microbial community within the respiratory tract, and any disturbance can be detrimental to the host. Answering such questions with more research across a range of chronic respiratory diseases will enable a deeper understanding of the mechanisms that drive microbial coexistence in mucus environments and hopefully facilitate the design of novel treatment approaches to alleviate mucosal, microbial, and mucus dysregulation.

## Figures and Tables

**Table 1:** Mucus parameters in pulmonary disease. Mucus solids concentration (%), total mucin, composition of MUC5B and MUC5AC and their ratio in asthma, non-CF bronchiectasis, CF, pulmonary eosinophilia, pulmonary alveolar proteinosis, and COPD.

Type of Comparison	Stratification	Mucus Parameters					Reference
		Mucus Solids Concentration (%)	Total Mucin µg/mL	MUC5B µg/mL or µg/g	MUC5AC µg/mL or µg/g	MUC5B: MUC5AC	
Asthma							
History of sputum production	Normal	-	800	-	-	-	[46]
	Asthma (no phlegm)	-	1100	-	-	-	
	Asthma (phlegm)	-	4250	-	-	-	
Pediatric asthma	Normal	-	246	239	7.6	7.3:1	[47]
	Stable asthma	-	231	208	22	9.33:1	
	Acute asthma	-	211	166	45	3.7:1	
Asthma phenotype	Normal	-	1597 (3101)	269 (276)	1328 (1030)	-	[48]
	Asthma	-	4753 (5785)	1798 (3130)	2955 (2720)	-	
Effect of asthma exacerbation	Normal	-	-	-	-	1.8:1	[49]
	Stable	-	-	-	-	0.7:1	
	Exacerbation	-	-	-	-	0.6:1	
Non-CF Bronchiectasis							
Phenotype	Normal	1.9	1820	85	3	28:1	[30]
	Bronchiectasis	2.7	4549	550	100	5.5:1	
Induction method	Induced	-	3800	-	-	-	[30]
	Spontaneous	-	4800	-	-	-	
*P. aeruginosa* infection	Positive	-	-	Below det.	6.9 × 10^−4^	-	[50]
	Negative	-	-	Below det.	4.7 × 10^−4^	-	
Cystic fibrosis (CF)							
Mucin hyperconcentration	Non-CF	-	2710	7.8	1.2	6.5:1	[51]
	CF	-	6454	1.1	1.1	1:1	
Mucus hyperconcentration	Adult CF	5.2 (2.3)	-	-	-	-	[52]
Mechanisms initiating pediatric lung disease	Non-CF pulmonary disease	-	229 (455)	-	-	3.7:1	[53]
	CF	-	475 (1089)	-	-	3.7:1	
Pulmonary eosinophilia (PE)							
Airway mucin concentration	Normal	-	-	-	8.0 × 10^−6^	-	[54]
	PE	-	-	-	2.0 × 10^−5^	-	
	Chronic PE	-	-	-	2.3 × 10^−5^	-	
Pulmonary alveolar proteinosis (PAP)							
Airway mucins in PAP	Normal	-	-	9.0 × 10^−5^	1.0 × 10^−5^	-	[55]
	PAP	-	-	5.4 × 10^−5^	9.0 × 10^−6^	-	
Chronic obstructive pulmonary disease (COPD)							
Annualized exacerbation rate	0	-	2200	-	-	-	[56]
	> 0 to < 2	-	2400	-	-	-	
	≥ 2	-	4200	-	-	-	
Sputum production	Never smoker	-	1600	-	-	-	[56]
	Ever smoker	-	2200	-	-	-	
	Ever smoker, phlegm	-	3100	-	-	-	
COPD severity (lung function)	Gold Stage 0	-	2400	-	-	-	[56]
	Gold Stage 1	-	2450	-	-	-	
	Gold Stage 2	-	3100	-	-	-	
	Gold Stage 3	-	3100	-	-	-	
Effect of smoking and a diagnosis of chronic bronchitis (CB)	Never smoker (age 27)	-	1603 (1041)	-	-	-	[56]
	Ever smoker (age 30)	-	1692 (827)	-	-	-	
	CB, ever smoker (age 61)	-	3868 (2297)	-	-	-	
Current or former smoker	Never smoker	1.96	1.84	-	-	-	[57]
	Ever smoker	1.7	1.45	-	-	-	
	CB, ever smoker	3	2.92	-	-	-	
				**MUC5B pmol/mL**	**MUC5AC pmol/mL**		
COPD symptom severity	Normal, never smoker	-	-	110	15	7.3:1	[56]
	No COPD, ever smoker	-	-	220	80	2.8:1	
	Mild-/Mod COPD, ever smoker	-	-	170	75	2.3:1	
	Severe COPD, ever smoker	-	-	300	110	2.7:1	
Diagnostic applicability	No CB	-	1500	95	8.6	11:1	[56]
	Classically defined	-	3500	200	52	3.8:1	
	SGRQ^ defined	-	3200	230	53	4.3:1	

-, value not measured; ~ (S.D.), standard deviation from the mean; ^ St. George’s Respiratory Questionnaire.

**Table 2:** Cumulative findings in the relevance of mucin properties to pulmonary disease states including asthma, non-CF bronchiectasis, CF, pulmonary eosinophilia, pulmonary alveolar proteinosis, and COPD.

Disease Type	Cumulative Findings
Asthma	Elevated sputum production is associated with increased mucin concentration [46] and changes to sputum structure (MUC5AC-tethering), which likely contributes to airway obstruction and mucus plugging [58]. In pediatric asthma, a higher ratio of MUC5AC to MUC5B is reported in both stable and acute asthmatic states vs. normal controls [47].
Non-CF bronchiectasis	Elevated sputum production with both MUC5AC and MUC5B, indicating increased mucus and mucin production compared to normal controls [30].
Cystic fibrosis	Airways exhibit mucin hyperconcentration [51] and corresponding impaired mucus clearance [52], which precedes structural change and infection in children with CF [53].
Pulmonary eosinophilia	Elevated levels of MUC5AC are observed but no difference between acute and chronic pulmonary eosinophilia are described [54].
Pulmonary alveolar proteinosis	While MUC5B is significantly reduced in patients with PAP compared to normal individuals, MUC5AC is comparable [55].
COPD	Airway mucin concentration is higher in current and former smokers, which represents a key component of the pathophysiology that mediates disease severity [56]. Both MUC5AC and MUC5B are elevated in current and former smokers with severe COPD, with an increased proportion of MUC5AC observed [56].

## Data Availability

Not applicable.
